# Assessing Brain Tissue Viability on Nonenhanced Computed Tomography After Ischemic Stroke

**DOI:** 10.1161/STROKEAHA.122.041241

**Published:** 2023-01-05

**Authors:** Awad Alzahrani, Xinyu Zhang, Adel Albukhari, Joanna M. Wardlaw, Grant Mair

**Affiliations:** 1 Centre for Clinical Brain Sciences, University of Edinburgh, United Kingdom (A. Alzahrani).; 2 Department of Diagnostic Radiology, Faculty of Applied Medical Sciences, King Abdulaziz University, Jeddah, Saudi Arabia (A. Alzahrani).; 3 School of Medicine, University of Dundee, United Kingdom (X.Z.).; 4 Department of Radiology, King Abdulaziz University Hospital, Jeddah, Saudi Arabia (A. Albukhari).; 5 Edinburgh Imaging, and UK Dementia Research Institute at the University of Edinburgh and Centre for Clinical Brain Sciences, University of Edinburgh, United Kingdom (J.M.W., G.M.).

**Keywords:** brain, ischemia, perfusion, stroke, tomography

## Abstract

Treatment for ischemic stroke can be offered beyond conventional time limits for patients with favorable computed tomography perfusion (CTP), but this is not universally available. We sought a threshold for brain attenuation on nonenhanced computed tomography (NECT) to differentiate CTP-defined penumbra vs core, and correlated NECT features with CTP.

**Methods:**

We retrospectively assessed consecutive patients presenting to King Abdulaziz University Hospital with ischemic stroke (2017–2020), baseline NECT, and a visible defect on concurrent CTP. Using CTP as the reference standard, we measured the attenuation of ischemic and healthy contralateral brain on NECT to produce attenuation ratios (ischemic/normal) for penumbra and core. We used area under the receiver operating characteristic curve to estimate the optimal computed tomography (CT) attenuation ratio for penumbra. Per patient, we qualitatively assessed 8 regions within the affected cerebral hemisphere: on NECT as normal, hypoattenuating (with/out swelling), or isolated swelling and on CTP as normal, penumbra, or core. We sought associations between isolated swelling and penumbra, and between hypoattenuation and core.

**Results:**

We include 142 patients (86 male), mean age 61±14 years. Median 261 minutes (interquartile range, 173–382) to NECT. We measured 206 ischemic lesions (124 penumbra, 82 core). Optimal CT attenuation ratio for identifying penumbra was >0.87, with 86% sensitivity 91% specificity (area under the receiver operating characteristic curve, 0.95 [95% CI, 0.92–0.98]; *P*<0.0001). We qualitatively assessed 976 cerebral regions (72 isolated swelling, 254 hypoattenuation). On NECT, isolated swelling usually corresponded to CTP penumbra (70/72, 97%), whereas visible NECT hypoattenuation was found with core (141/254, 56%) and penumbra (109/254, 43%). CTP core lesions were rarely normal on NECT (13/155, 8%).

**Conclusions:**

After ischemic stroke, brain tissue viability can be assessed using NECT. Isolated swelling is highly specific to penumbra. Visible hypoattenuation does not always represent core, nearly half of such lesions were penumbral on concurrent CTP and can be differentiated by measuring lesion attenuation.

For patients with delayed presentation acute ischemic stroke or where time of symptom onset is unknown, randomized-controlled trials have shown that both endovascular therapy and intravenous alteplase remain effective and can be safely administered as late as 24 hours after symptom onset.^1–3^ Patient selection for recruitment into these trials was largely based on detection of ischemic rather than infarcted regions as determined by advanced imaging techniques such as computed tomography perfusion (CTP) or magnetic resonance imaging (MRI).^1,2^ The 2019 update to American Heart Association guidelines now recommend advanced imaging to distinguish core from penumbra in extended time windows, last known well, or wake-up patients with acute ischemic stroke.^4^ However, only 53% of stroke centers globally routinely apply these advanced imaging recommendations,^5^ presumably limited by relevant local experience and resources. Thus, there is a desperate need for other, perhaps simpler imaging methods in clinical practice to help guide acute treatment decision-making for ischemic stroke.

Even when time of symptom onset is known and is within accepted limits for treatment, another key selection criterion, especially for thrombectomy and in centers without access to more advanced imaging, is the detection of ischemic features in brain on nonenhanced computed tomography (NECT) such as parenchymal hypoattenuation or swelling. American Heart Association guidelines recommend thrombectomy for treating large vessel occlusion stroke in patients with an Alberta Stroke Program Early CT Score (ASPECTS) ≥6.^6^ This is because most major thrombectomy trials excluded patients with ASPECTS <6 on the basis they likely have a large infarct core, and thus poor prognosis, even with thrombectomy.^7^ However, ASPECTS does not differentiate between brain tissue hypoattenuation and swelling, despite evidence these features may reflect different stages of ischemia, even tissue viability.^8,9^

Over minutes to hours, the ischemia-infarction cascade of brain is associated with increasing edema.^10^ Computed tomography (CT) scanners are calibrated to the attenuation coefficient of water,^11^ and can thus detect even very small differences in the water content of brain as a reduction in tissue attenuation measured in Hounsfield units (HU). Very early ischemic changes (within minutes of stroke onset) induce cytotoxic edema, but this should not affect the CT attenuation of brain because there is no net water uptake.^12^ However, within hours from stroke onset, subtle changes can be identified as parenchymal hypoattenuation or tissue swelling secondary to progressive ionic edema, and a net uptake of water.^12^ In vitro measurements showed a relationship between CT attenuation and water content: using a gel medium, a 1% increase in water content caused a decrease of 2.6 HU.^13^

Among patients with ischemic stroke, reduced brain attenuation on NECT is associated with corresponding abnormalities on other imaging modalities including diffusion-weighted MRI, positron emission tomography, and perfusion imaging.^14,15^

There is, however, uncertainty in how early ischemic features on NECT translate to the different pathophysiological processes of acute ischemic brain injury. Specifically, whether NECT can be used to differentiate reversible from irreversible ischemic injury in the acute stroke phase, when treatment decisions are urgent and when other imaging modalities may be unavailable.

The aims of our study are first, to determine whether a specific NECT brain attenuation threshold exists to differentiate reversible (penumbra) and irreversible (core) ischemic brain tissue injury, as defined using CTP; and second, to correlate visually appreciable early ischemic features on NECT with concurrent CTP changes indicative of penumbra or core.

## Methods

### Patient Selection

We retrospectively reviewed data from all patients who presented with acute symptoms of stroke to King Abdulaziz University Hospital, Jeddah, Saudi Arabia, between January 1, 2017, and December 31, 2020. Patient data were screened consecutively using predetermined inclusion criteria: (1) acute ischemic stroke presentation within 24 hours of symptom onset; (2) known time of symptom onset; (3) complete baseline NECT, and concurrent CT perfusion; (4) visually evident brain ischemia as indicated by CT perfusion imaging (but not necessarily visible on NECT). Exclusion criteria: (1) patients with intracranial hemorrhage, old stroke lesions, or other alternative structural causes for stroke symptoms; (2) nondiagnostic or missing CTP data; (3) abnormality on both sides of the brain. Baseline clinical characteristics (including National Institutes of Health Stroke Scale [NIHSS]) and demographic information was extracted from the medical records. We report our results according to Standards for the Reporting of Diagnostic Accuracy Studies criteria.^16^

The data that support the findings of this study are available from the corresponding author upon reasonable request.

### Study Approval and Patient Consent

The study was approved by the National Biomedical Ethics Committee at King Abdulaziz University (HA-02-J-008) in accordance with ethical guidelines. The committee waived the need for informed consent for this retrospective analysis.

### CT Acquisition

CT examinations were performed using 2 multidetector scanners (128 and 64 slice SOMATOM Definition AS; Siemens Healthcare, Germany). Whole-brain NECT: 120kV, 400 mA, 2.5 mm slice thickness, scan type was axial, 1-second rotation time. This was immediately followed by CTP: 80 kVp and 200 mA. CTP comprised 2 simultaneously acquired 40-second series of 1 image per slice per second, commencing 5 seconds after intravenous administration of 45 mL nonionic iodinated contrast at a rate of 4 mL/second via a power injector. Each perfusion series covers a 24mm axial section acquired as 2 adjacent 12mm slices with no gap. The first section was at the level of the basal ganglia/ internal capsule, the second was placed directly above, toward the vertex. CTP data were processed using the manufacturer’s software (Syngo Neuro Perfusion CT; Siemens Healthineers, Forchheim, Germany) to generate standard perfusion maps: cerebral blood volume (CBV), cerebral blood flow (CBF), mean transit time (MTT).

### Image Viewing and CTP Definitions

A commercially available Sectra workstation (IDS7, Version 24.1.2.5320, Linkoping, Sweden) was used to view all CTs in an ambient light environment using standard brain (center 35 HU, width 70 HU) and/or narrowed “stroke” (center 25–35 HU, width 30–40 HU) window settings for NECT, adjusted according to user preference. CTP was viewed using standard color maps. We defined CTP core, as visibly matched areas of decreased CBF and CBV with increased MTT; and CTP penumbra as visibly decreased CBF with maintained or increased CBV, and increased MTT.

### Quantitative Assessment of Imaging: Measuring CT Attenuation

**Figure 1.**
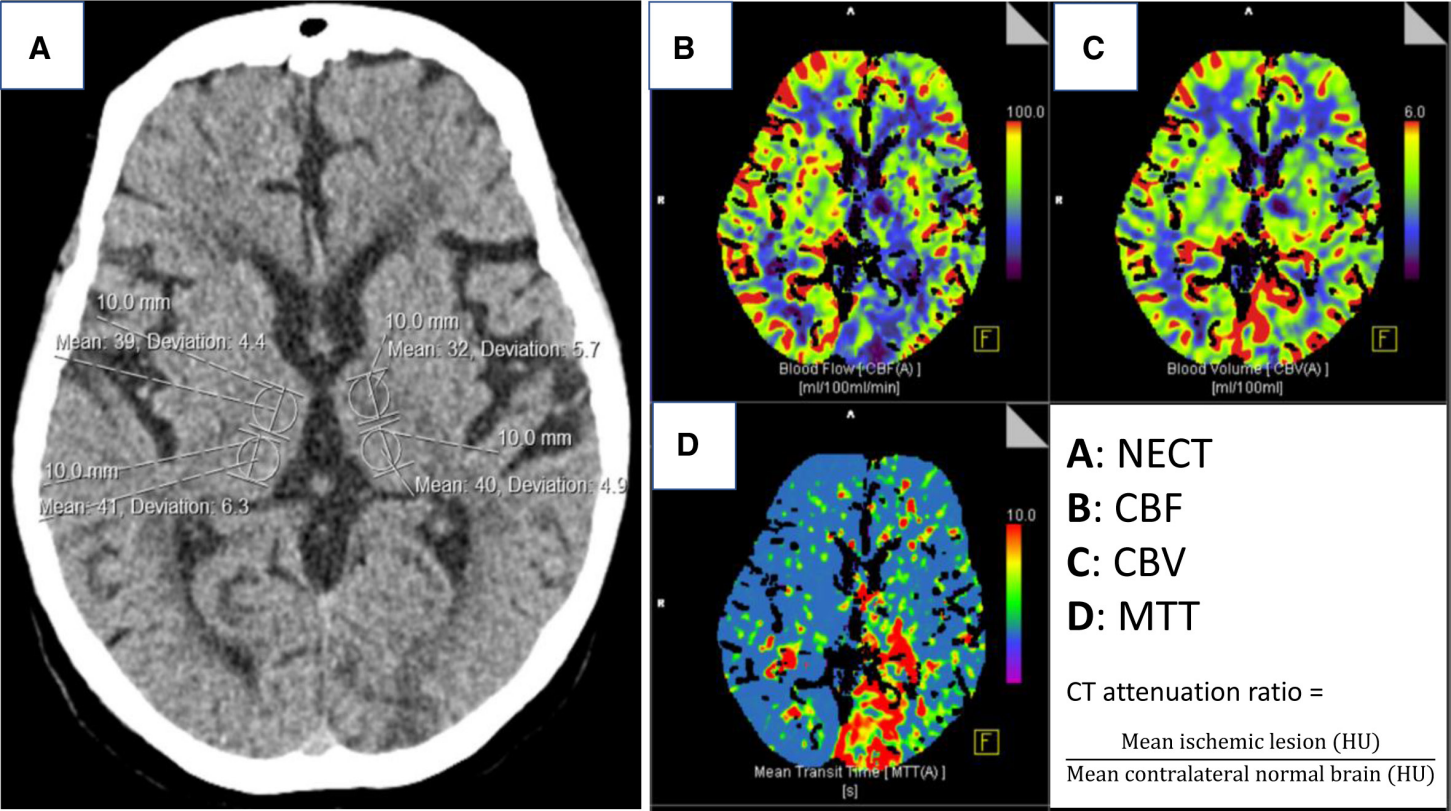
**Measuring nonenhanced computed tomography (NECT) attenuation ratio for core and penumbral lesions in a 60-year-old patient with left-sided ischemia.** NECT with measurements (**A**) and coregistered concurrently acquired computed tomography (CT) perfusion maps (**B–D**) demonstrate areas of lesion core (decreased cerebral blood flow [CBF; **B**] and cerebral blood volume (CBV; **C**) with increased mean transit time (MTT; **D**) and penumbra (decreased CBF with maintained or increased CBV and increased MTT). On NECT (**A**): **left upper** ROI measured CT attenuation of core lesion (32 Hounsfield units [HU]) mirrored to corresponding normal right hemisphere (39 HU), attenuation ratio 0.82; **left lower** ROI measured CT attenuation of penumbral lesion (40 HU) mirrored to corresponding normal right hemisphere (41 HU), attenuation ratio 0.98.

NECT attenuation of core and penumbral brain lesions was assessed (by a single reader with 2 years of experience assessing stroke imaging) using manually applied regions of interest (ROI) within each lesion type and within contralateral normal brain mirrored on the sagittal midline (Figure 1). To guide attenuation measurements of NECT, we used concurrently acquired CT perfusion imaging (viewed in parallel and coregistered using the Sectra Automatic Registration tool) to identify brain areas belonging to core or penumbral lesions using the CTP definitions described above. For all measurements, the standard Sectra Oval ROI measurement tool was used, and where possible, a 100 mm^2^ area round or ovoid ROI was applied. Smaller ROI were used if required (eg, to avoid focal calcification or cerebrospinal fluid spaces). Zoomed images were used to ensure accurate placement of ROIs. The mean HU value for each ROI was recorded. We derived attenuation ratios for core and penumbral lesion measurements as previously described (ischemic lesion attenuation ÷ normal brain attenuation).^11^

#### Reader Reliability for CT Attenuation Measurement

Reader-reliability assessment for quantitative image analysis was performed using a random sub-sample of our study cohort assessed separately. A second reader, masked to all imaging and clinical data (including previous ROI placement and results) and with over 10 years of experience assessing ischemic stroke lesions on CT, used the same method described above and independently scored 22 baseline NECT scans from 22 patients. We assessed interrater agreement for mean ROI results and attenuation ratio.^17^

### Qualitative Assessment of Imaging: Visual Scoring of Ischemic Features on NECT and CTP

**Figure 2.**
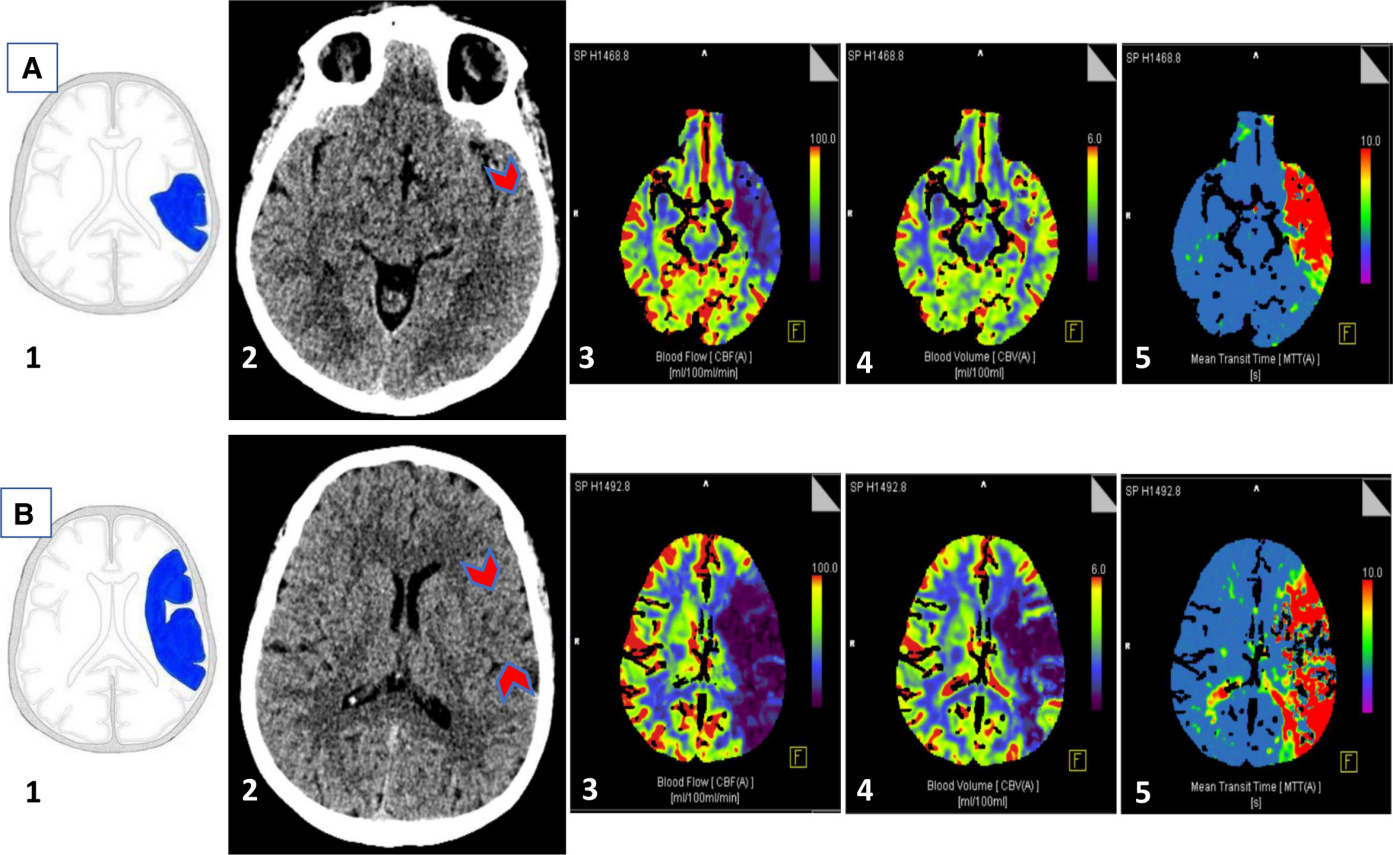
**Example computed tomography (CT) scan assessment of 2 brain regions from the IST-3 trial (Third International Stroke Trial) template for the same patient. A**, (1) Posterior half of peripheral middle cerebral artery (MCA) territory, (2) isolated focal swelling on nonenhanced CT (NECT), (3) decreased cerebral blood flow (CBF), (4) normal cerebral blood volume (CBV), and (5) increased mean transit time (MTT). **B**, (1) most of peripheral MCA territory + lateral part of basal ganglia, (2) subtle parenchymal hypoattenuation on NECT, (3) decreased CBF, (4) decreased CBV, (5) increased MTT.

Each baseline NECT scan was evaluated independently by 2 reviewers. Major arterial territories of brain were scored individually using the IST-3 trial (Third International Stroke Trial) ischemia score,^18^ which divides the brain into regions for assessment. We used 8 regions per cerebral hemisphere. Five regions within the middle cerebral artery territory [(1) basal ganglia, (2) white matter lateral to lateral ventricle, (3) anterior half of peripheral middle cerebral artery territory, (4) posterior half of peripheral middle cerebral artery territory, (5) lateral part of basal ganglia], and one region for each of the anterior and posterior cerebral artery territories, and border zones (anterior and posterior; Figure S1). This location-based method simplifies assessment of scans with only faintly visible lesions on NECT alone. Each brain region on NECT was scored as normal, visible parenchymal hypoattenuation with or without swelling or isolated tissue swelling (without visible hypoattenuation). Parenchymal hypoattenuation was defined as a region of visibly decreased attenuation when compared to other parts of the same structures or the opposite hemisphere.^19^ Reviewers were careful to ensure hypoattenuating ROI had a distribution compatible with ischemic stroke, rather than leukoaraiosis (eg, for stroke, wedge-shaped involving cortex and white matter). Isolated swelling was defined by asymmetric sulcal effacement in the absence of cortical hypoattenuation (ie, cortex and subjacent white matter remain distinguishable) or other causes for mass effect, for example, tumor.^20^ At least 2 weeks later and blinded to other patient data including NECT findings, each corresponding CTP scan was assessed using the same 8 regions as above. Individual brain regions were compared with equivalent contralateral brain using the definitions for core and penumbra defined above (Figure 2). Disagreement between reviewers for the most appropriate visual score was decided by consensus, that is, we did not assess reader agreement for this component.

### Statistical Analysis

NIHSS was categorized into 4 groups (0–4 No to Minor stroke; 5–15 Moderate stroke; 16–20 Moderate severe stroke; 21–42 Severe stroke).^21^ The Kolmogorov-Smirnov Test was used to assess normality of continuous data, and mean (m), SD reported. We report median (M) and interquartile range for variables that were not normally distributed. Categorical variables are reported as frequencies and proportions. We used independent-sample *T* tests to compare means, Mann-Whitney *U* tests to compare medians. The area under the receiver operating characteristic curve was used to estimate the optimal CT attenuation ratio cutoff for distinguishing penumbra from core. Inter-rater agreement of quantitative measures was assessed using intraclass correlation coefficient (ICC) estimates (and their 95% CI) based on a single rating, absolute agreement, 2-way random effects model suitable for continuous data. We used cross-tabulation to investigate associations between swelling and penumbra, hypoattenuation, and core. A Fisher exact test was used to seek associations between tissue swelling and penumbra and between hypoattenuation and core. Fisher exact test was used instead of a χ^2^ test, as one or more of the cell counts in a 2×2 table was <5. Statistical analyses were conducted using SPSS V24.0 (IBM‚ SPSS Statistics). Alpha level was set at 0.05/ test number to determine 2-tailed significance but we preferentially report 95% CI where possible.

## Results

**Figure 3.**
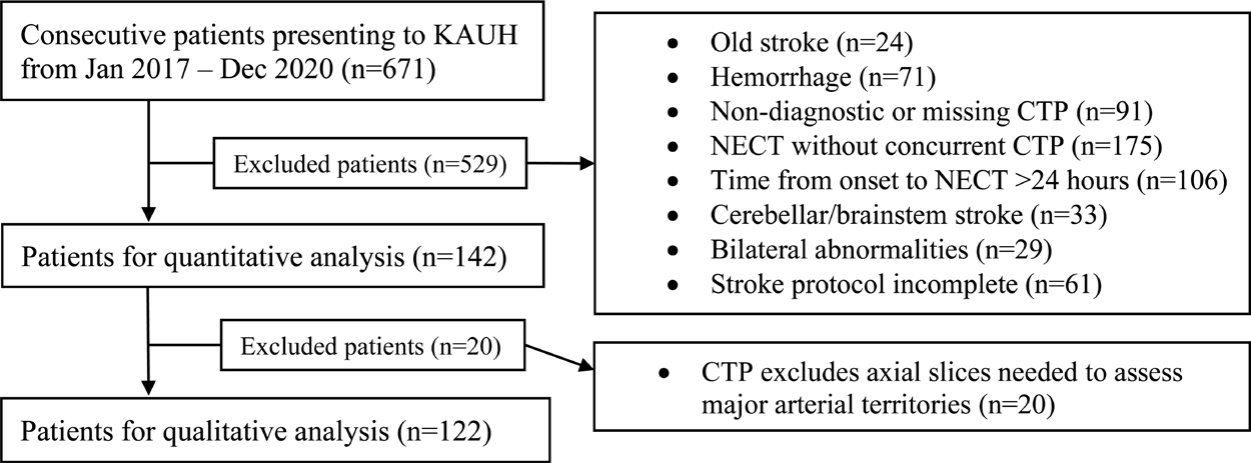
**Flowchart for patient selection.** CTP indicates computed tomography perfusion; KAUH, King Abdulaziz University Hospital; and NECT, nonenhanced computed tomography.

Within the study period, data for 671 consecutive patients were retrieved from the hospital database, with 529 exclusions after screening. Finally, 142 patients met all inclusion criteria. However, 20 of these 142 patients were excluded from the qualitative analysis due to incomplete CT perfusion field of view (Figure 3).

Of 142 patients, 86 (60.6%) were male; mean age was 61.5 years, SD, 14.7 years; most patients (nearly 60%) had moderate stroke (NIHSS, 5–15), <6% of participants had severe stroke (NIHSS, 21–42). Patient characteristics are listed in Table 1.

**Table 1.**
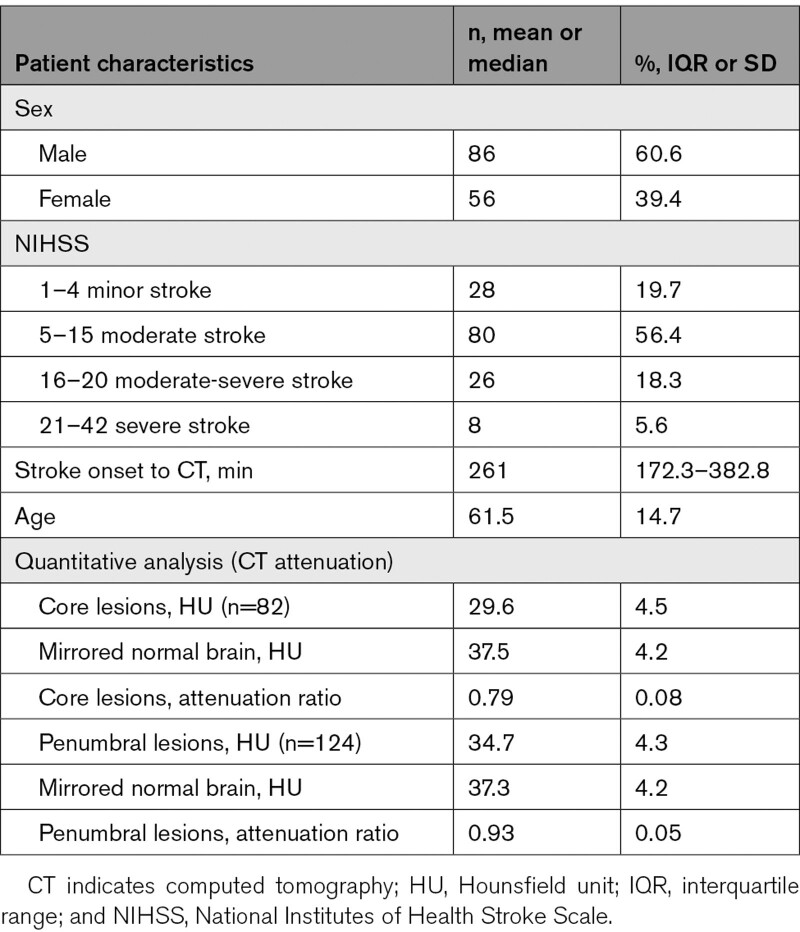
Demographic and CT Characteristics of the Study Population (n=142)

### Measuring CT Attenuations Changes (Quantitative Assessment)

From 142 CT scans assessed, we measured 206 ischemic lesions; 124 penumbra, and 82 core. There were 64 patients with both lesion types, 60 patients with penumbral lesions, and 18 patients with core lesions. The mean penumbral lesion attenuation was 34.7 (SD, 4.3) HU, compared to a mean attenuation of 37.3 (SD, 4.2) HU in contralateral normal brain. Thus, the mean penumbral attenuation ratio was 0.93. The mean core lesion attenuation was 29.6 (SD, 4.5) HU, compared to a mean attenuation of 37.5 (SD, 4.2) HU in contralateral normal brain. Thus, the mean core attenuation ratio was 0.79. There was a statistically significant increase in the mean CT attenuation ratio of penumbral lesions compared with core lesions of 0.14, t(131.78)=14.43, *P*<0.001 (Table S1).

Interrater agreement for the assessment of core lesions, contralateral normal brain, and attenuation ratio was ICC (95% CI) 0.92 (0.77–0.97), 0.76 (0.68–0.97), and 0.81 (0.56–0.93), respectively. Interrater agreement for the assessment of penumbral lesions, contralateral normal brain, and attenuation ratio was ICC (95% CI) 0.73 (0.43–0.88), 0.68 (0.35–0.86), and 0.70 (0.22–0.88), respectively.

**Figure 4.**
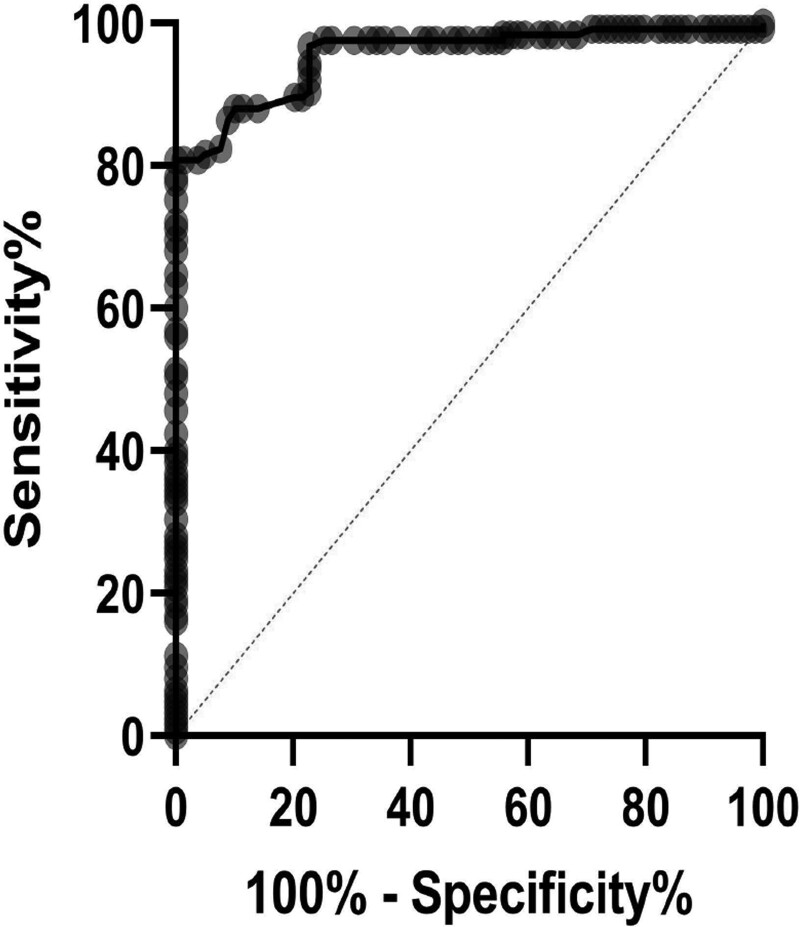
**Receiver operating characteristic analysis testing the expected discriminative ability of the optimal attenuation ratio (>0.87) to distinguish penumbra from core on baseline nonenhanced computed tomography.** Area under curve 0.955.

Receiver operating characteristic analysis of all 206 ischemic lesion measurements suggested an attenuation ratio >0.87 would provide the optimal test sensitivity of 86.4% (95% CI, 79.3%–91.3%) and specificity of 91.1% (95% CI, 82.8%–95.6%), for correctly identifying penumbral lesions. Area under the curve for receiver operating characteristic analysis was 0.955 (95% CI, 0.929–0.981; Figure 4).

### CT Features of Ischemia and Perfusion Changes (Qualitative Assessment)

We qualitatively assessed 976 cerebral regions from 122 acute ischemic stroke patients. There were 72 regions assessed as isolated tissue swelling on NECT, 70 were penumbra, with only 1 normal and 1 with core appearances on CTP. Of 254 regions assessed as visible parenchymal hypoattenuation (with/out swelling) on NECT, 141 were core, 109 had penumbral appearances on CTP. Core lesions on CTP were rarely normal on NECT (13/155; Table 2).

**Table 2.**
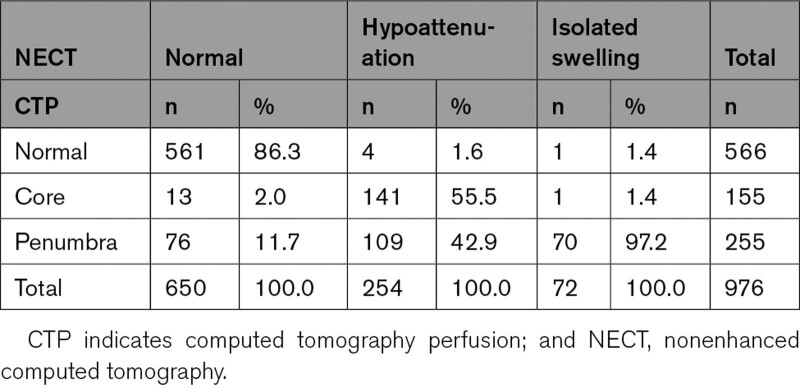
Proportion of Regions Having Perfusion Characteristics of Core, Penumbra, and Normal Tissue According to Qualitative Analysis

There was a statistically significant difference in the proportions of regions with normally perfused tissue, core, and penumbra relative to the NECT appearances (χ^2^=827.16, df=4, *P*<0.001) with a higher proportion of core pattern in visible hypoattenuation and a higher proportion of penumbra in regions with isolated swelling (Table 2).

On Fisher exact testing, relationships between swelling and penumbra and between visible hypoattenuation and core were significant, *P*<0.001 for both.

## Discussion

We demonstrate that CT attenuation measurements on NECT enable differentiation of penumbra and core brain tissue injury in acute ischemic stroke, as defined using concurrent CT perfusion imaging. We used the ratio of manually measured CT attenuation in ischemic brain relative to contralateral normal brain. This ratio approach is rapid with high accuracy and reasonable precision to identify brain regions that are affected by ischemic hypoperfusion demonstrated on CT perfusion, with an area under the receiver operating characteristic curve up to 0.95. The optimal CT attenuation ratio for distinguishing penumbra from core was 0.87. Our simple NECT ratio approach provides quantitative data and therefore, should be more reproducible than visual assessment alone. Our method might eventually be useful in clinical practice to assist clinicians evaluate NECT imaging in patients with a prolonged or unknown stroke onset time who are still considered potential candidates for endovascular therapy or thrombolysis but where more advanced imaging such as CTP is unavailable, although this requires prospective testing. In addition, we anticipate these results may be useful to develop in-depth automated software analysis for patient-specific ischemic lesion measurements on NECT.

Among patients with delayed or unknown time of ischemic stroke onset, eligibility for endovascular thrombectomy in the DAWN (DWI or CTP Assessment With Clinical Mismatch in the Triage of Wake-Up and Late Presenting Strokes Undergoing Neurointervention With Trevo) and DEFUSE 3 (Endovascular Therapy Following Imaging Evaluation for Ischemic Stroke) trials and for intravenous thrombolysis in the EXTEND trial (Extending the Time for Thrombolysis in Emergency Neurological Deficits) was largely based on determination of ischemia (rather than infarct) using CTP or MRI.^1,3,22^ This poses a challenge as access to CTP or acute MRI is not routinely available in many stroke centers around the globe. In centers without access to these more advanced imaging modalities, such as those in developing countries or even in small perhaps remote, nonspecialist hospitals in more advanced healthcare systems, simpler methods to identify patients for treatment using routine imaging (like nonenhanced CT) are required.

Our CT attenuation ratio approach is simple and provides a NECT imaging surrogate of CT perfusion-defined penumbral and core lesions. A prior analysis comparing early ischemic changes on NECT and CT perfusion in patients who underwent NECT, CTP, and MRI within 100 minutes showed that NECT ASPECTS was not inferior in determining CTP ischemic core and in identifying an MRI diffusion ischemic lesion.^23^ However, intrarater and inter-rater variability, the time delay between modalities, and small sample sizes might limit the general applicability of that approach. In 41 patients undergoing thrombectomy, Mokin et al^24^ estimated that attenuation ratios of 0.94 to 0.96 within ASPECTS regions at baseline correlated best with 24-hour ASPECTS. However, they measured larger areas of brain (ASPECTS regions) that are more likely to include both ischemic and normal tissue, and the 24-hour delay to assess core means some baseline penumbra might be lost. Both differences are likely to raise their penumbra-core threshold closer to 1. Another study demonstrated that baseline NECT attenuation is associated with development of cerebral hemorrhage (and thus tissue death) in ischemic stroke patients receiving intravenous alteplase.^25^ Although implementation of CTP is possible on most CT scanners, it inevitably causes delays and yet time to treatment remains a predictor of poor outcome in ischemic stroke.^26,27^ CT perfusion imaging may add around 15 minutes to thrombectomy decision-making.^28^ By comparison, our CT attenuation ratio approach is likely to be faster.

Reader reliability for our method (measuring CT attenuation changes) was good to excellent. We found high levels of agreement for core lesion measurement between 2 readers (ICC, 0.92) but slightly less agreement for penumbral lesion measurement (ICC, 0.73). Interestingly, interrater agreements were slightly less for attenuation measurement of the contralateral normal brain (ICC, 0.76, 0.68, respectively), but these did not significantly negatively impact agreement for the calculated attenuation ratio of core (ICC, 0.81) and penumbra (ICC, 0.70). These results are encouraging considering one of readers had over 10 years’ of experience assessing stroke CT, the other only 2 years.

A second finding of our analysis is that visible parenchymal hypoattenuation and isolated tissue swelling are associated with different pathophysiological subtypes of brain injury. Specifically, regions with isolated tissue swelling had increased/or normal CBV, reduced CBF, MTT, and are indicative of penumbral tissue. Regions with visible parenchymal hypoattenuation were associated with reduced CBV, CBF, and increased MTT, which are indicative of core.

Our study extends knowledge about the pathophysiology of early ischemic features.^8,9^ A previous study suggested that isolated brain tissue swelling was correlated with increased cerebral blood volume on MR perfusion, but there was delay between NECT and MRI in most patients.^20^ Muir et al demonstrated that focal swelling on NECT corresponded with increased cerebral blood volume on CT perfusion and with perfusion parameters of penumbral tissue.^29^ In our study, isolated tissue swelling (without hypoattenuation) was almost exclusively found in those with penumbral perfusion (only 1 patient with CTP core had isolated swelling). This swelling may represent compensatory vasodilation after a mild decrease of cerebral blood flow or early postischemic hyperperfusion.^8^

Conversely, visible parenchymal hypoattenuation after ischemic stroke is likely due to brain tissue edema,^13^ reflecting severe, possibly irreversible tissue injury.^12,30^ An acute CBF reduction induces ischemic edema and thus parenchymal hypoattenuation.^12^ Indeed, we found that visible parenchymal hypoattenuation was associated with reduced CBV and CBF and elevated MTT, that is. presumed tissue core. However, a substantial number of visibly hypoattenuating regions in our analysis had penumbral perfusion on CTP (109/254, 43%), suggesting that hypoattenuation does not always reflect infarction, although affected tissue might be at higher risk of progressing to infarction.

This then generates debate on whether early treatment can halt, or possibly reverse edema caused by ischemic injury. In a previous study involving 786 patients with acute ischemic stroke and using follow-up CT scan as a reference, the positive predictive value for early parenchymal hypoattenuation was 96%, suggesting that obvious hypoattenuation is most likely to represent irreversible tissue damage.^31^ Using rats, another study attempted to investigate the irreversibility of early vasogenic edema.^32^ It was observed that although reperfusion was swiftly achieved, recovery from ischemic edema was usually not possible. In 4 rats (7.5%), however, attenuation first decreased but subsequently came back to normal. The authors suggested that although hypoattenuation due to early ischemic water uptake may be highly indicative of irreversibly brain tissue injury, it may still be modulated by timely reperfusion.^32^ Other animal studies have suggested that some net water uptake occurs in ischemic brain tissue (ionic edema) before cell death,^33^ but the interval between the two may be short.

### Strengths and Limitations of the Present Work and Future Plans

We used concurrent advanced imaging (acquired soon after baseline NECT) as the gold standard to minimize discrepancy caused by reversibility of early acute ischemic stroke features in the interval between the 2 imaging modalities, and to accurately measure NECT attenuation of core and penumbra, particularly in very early ischemic lesions that were not yet visible to the human eye (generally within the first hour—although we did not specifically record this). Although this approach, therefore, allowed us to robustly develop our method for a range of brain tissue injury types, the overarching aim of this work is to develop a method that can be used in the absence of CTP. We have separately found that NIHSS score >11 and a nonlacunar syndrome effectively identifies patients likely to have a medium-large ischemic lesion (and thus differentiates those with a nonvisible lesion from those where a lesion is simply too small to see).^34^ Additionally, the presence of a hyperattenuating artery can signpost users to the location of subtle ischemic lesions.

We deliberately used small ROI for attenuation measurement to limit the impact of CT volume averaging on results. To enable assessment of larger brain regions, multiple ROI may need to be applied but this allows for a better understanding of core-penumbral lesion heterogeneity. All CT attenuation measurements were retrospectively derived from the same manufacturer’s CT scanners. However, regarding cross-vendor performance, we assume a general translatability as only relative attenuation values and not absolute values were used for the analysis. We are planning prospective testing using a range of vendor hardware to increase the generalisability of our NECT attenuation ratio approach, to validate its use in real-time, and to determine the impact on treatment decision-making and outcome.

We used qualitative methods to assess CTP and thus define core and penumbra which are simple and fast but may be prone to reader variability. However, although automated thresholds would have reduced measurement variability, such an approach would still be liable to the technical heterogeneities implicit in CTP. We were limited to 3 parameter maps which may reduce the generalisability of our results, but there remains no firm consensus on which parameter maps and thresholds to use.^35,36^ There is a risk of selection bias in our study because we excluded a large number of patients with image artifacts, prior infarcts, hemorrhage, or bilateral abnormalities which would have reduced the accuracy of CT attenuation ratios.

Potential users of our method should remain aware of the biases and challenges resulting from, for instance, patient motion artifacts, or contralateral abnormality which may confound the association between relative CT attenuation value (HU) measurements and the acute ischemic lesion.

## Conclusions

In acute ischemic stroke, NECT may provide information on brain tissue viability. We demonstrated that CT attenuation measurements of brain can be used to differentiate between penumbral and core lesions as defined using concurrent CTP. Although visibly hypoattenuating regions on NECT are commonly associated with core, we found 42% were still viable according to CTP. Isolated brain tissue swelling is a highly specific marker of penumbra. These methods may improve patient selection for stroke treatment at extended time points, especially where imaging resources are limited but require prospective testing.

## Article Information

### Acknowledgments

For open access, the authors have applied a CC-BY public copyright license to any Author Accepted Manuscript version arising from this submission.

### Sources of Funding

Mr Alzahrani received a PhD scholarship from King Abdulaziz University, Saudi Arabia (2019–2023). Dr Wardlaw is part funded by the UK Dementia Research Institute Ltd which receives its funding from the UK Medical Research Council, Alzheimer’s Society, and Alzheimer’s Research UK. Dr Mair is supported by the Stroke Association Edith Murphy Foundation Senior Clinical Lectureship (SA_L-SMP_18\1000).

### Disclosures

None.

### Supplemental Material

Figure S1

Table S1

STARD Checklist

## Supplementary Material
