# Supplementary material for: Assessing Brain Tissue Viability on Nonenhanced Computed Tomography After Ischemic Stroke
Source: Stroke. 2023 Jan 5;54(2):558–66. doi: 10.1161/STROKEAHA.122.041241 (PMC9855746; doi:10.1161/STROKEAHA.122.041241)
Supplement: Supplementary file 1 [file str-54-558-s001.pdf]

SUPPLEMENTAL MATERIAL

Assessing brain tissue viability on non-enhanced CT after ischemic stroke

Awad Alzahrani (MSc)[1,2]; Xinyu Zhang (MPH)[3]; Adel Albukhari (MD)[4]; Joanna M. Wardlaw (MD)[5]; Grant Mair (MD)[5].

1. Centre for Clinical Brain Sciences, University of Edinburgh, UK
2. Department of Diagnostic Radiology, Faculty of Applied Medical Sciences, King Abdulaziz University, Jeddah, Saudi Arabia.
3. School of Medicine, University of Dundee, UK
4. Department of Radiology, King Abdulaziz University Hospital, Jeddah, Saudi Arabia.
5. Edinburgh Imaging, and UK Dementia Research Institute at the University of Edinburgh and Centre for Clinical Brain Sciences, University of Edinburgh, UK

Corresponding Author:

Dr Grant Mair
Centre for Clinical Brain Sciences
University of Edinburgh
Chancellor's Building
49 Little France Crescent
Edinburgh
EH16 4SB, UK

Phone: +44 1314659563
Email: grant.mair@ed.ac.uk

Figure S1: Templates for site classification of ischemic lesions on CT scan. (1) basal ganglia ischemic lesion, (2) ischemic lesion of white matter lateral to lateral ventricle, (3) ischemic lesion of anterior half of peripheral middle cerebral artery (MCA) territory, (4) ischemic lesion of posterior half of peripheral MCA territory, (5) ischemic lesion of entire peripheral MCA territory and lateral part of basal ganglia, (6) ischemic lesion of anterior cerebral artery territory, (7) ischemic lesion of posterior cerebral artery territory, (8) border zone ischemic lesions.

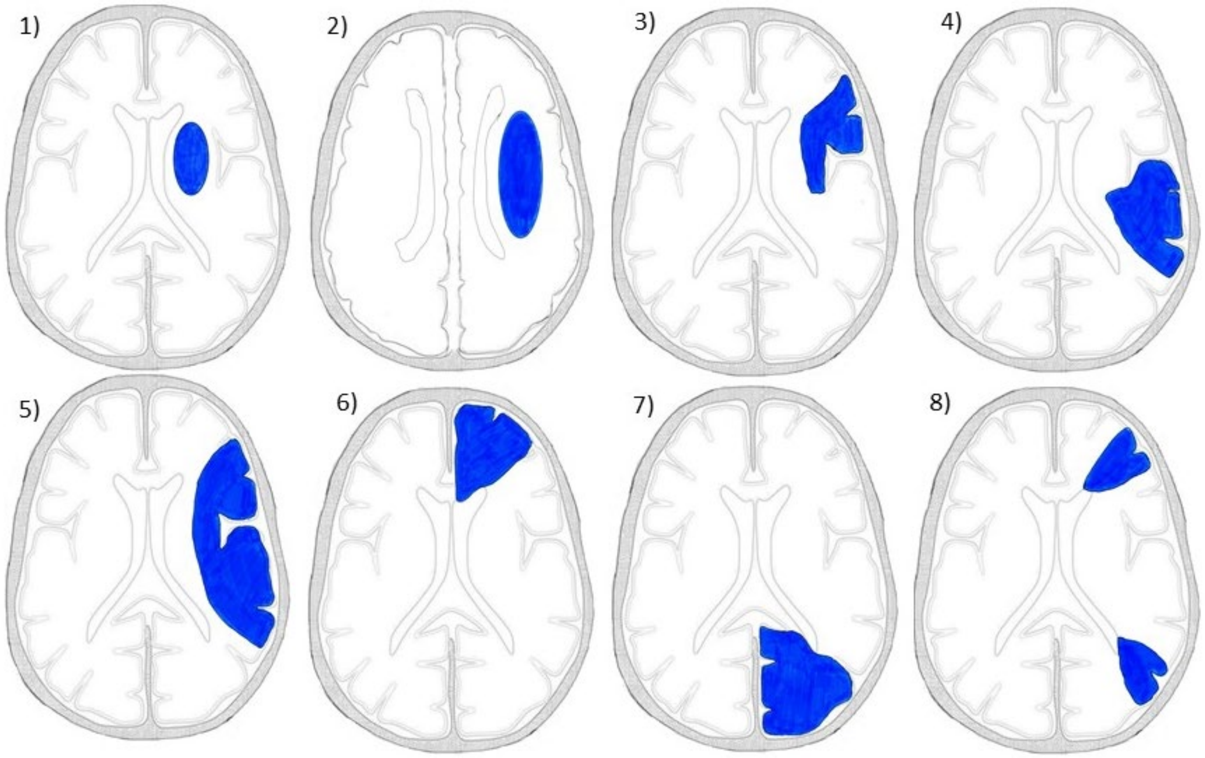

Note

Adapted, and used with permission of the American Society of Neuroradiology, from: A simple practical classification of cerebral infarcts on CT and its interobserver reliability. Wardlaw JM, Sellar R. *AJNR.* 15(10):1933-1939. 1994; permission conveyed through Copyright Clearance Center, Inc.

Table S1. Comparison of core CT attenuation ratio and penumbral CT attenuation ratio.

Ratio	N~	df*	t-value	p	mean	Δmean	95% CI of Δmean
Penumbral ratio	124	131.78	14.43	<0.001	0.93	0.14	1.58 to 2.32
Core ratio	82				0.79		

Note

~N, number of lesions; *df, degree of freedom; Δmean, the difference between the means in each group; F-test found unequal sample variances

STARD Checklist

Section & Topic	No	Item	Reported on page #
TITLE OR ABSTRACT			
	1	Identification as a study of diagnostic accuracy using at least one measure of accuracy (such as sensitivity, specificity, predictive values, or AUC)	1
ABSTRACT			
	2	Structured summary of study design, methods, results, and conclusions (for specific guidance, see STARD for Abstracts)	1, 2
INTRODUCTION			
	3	Scientific and clinical background, including the intended use and clinical role of the index test	4, 5
	4	Study objectives and hypotheses	5
METHODS			
Study design	5	Whether data collection was planned before the index test and reference standard were performed (prospective study) or after (retrospective study)	6
Participants	6	Eligibility criteria	6
	7	On what basis potentially eligible participants were identified (such as symptoms, results from previous tests, inclusion in registry)	6
	8	Where and when potentially eligible participants were identified (setting, location and dates)	6
	9	Whether participants formed a consecutive, random or convenience series	6
Test methods	10a	Index test, in sufficient detail to allow replication	6, 7
	10b	Reference standard, in sufficient detail to allow replication	6, 7
	11	Rationale for choosing the reference standard (if alternatives exist)	7, 8
	12a	Definition of and rationale for test positivity cut-offs or result categories of the index test, distinguishing pre-specified from exploratory	7-9
	12b	Definition of and rationale for test positivity cut-offs or result categories of the reference standard, distinguishing pre-specified from exploratory	7-9
	13a	Whether clinical information and reference standard results were available to the performers/readers of the index test	8, 9

	13b	Whether clinical information and index test results were available<br>to the assessors of the reference standard	8, 9
Analysis	14	Methods for estimating or comparing measures of diagnostic accuracy	9
	15	How indeterminate index test or reference standard results were handled	
	16	How missing data on the index test and reference standard were handled	10
	17	Any analyses of variability in diagnostic accuracy, distinguishing pre-specified from exploratory	9
	18	Intended sample size and how it was determined	
RESULTS			
Participants	19	Flow of participants, using a diagram	10
	20	Baseline demographic and clinical characteristics of participants	10
	21a	Distribution of severity of disease in those with the target condition	10
	21b	Distribution of alternative diagnoses in those without the target condition	
	22	Time interval and any clinical interventions between index test and reference standard	10
Test results	23	Cross tabulation of the index test results (or their distribution)<br>by the results of the reference standard	10, 11
	24	Estimates of diagnostic accuracy and their precision (such as 95% confidence intervals)	11
	25	Any adverse events from performing the index test or the reference standard	
DISCUSSION			
	26	Study limitations, including sources of potential bias, statistical uncertainty, and generalisability	14-16
	27	Implications for practice, including the intended use and clinical role of the index test	16
OTHER INFORMATION			
	28	Registration number and name of registry	
	29	Where the full study protocol can be accessed	
	30	Sources of funding and other support; role of funders	17
